# Ligament reconstruction with modified suture anchor fixation technique for chronic distal radioulnar joint instability: A case report and literature review

**DOI:** 10.1016/j.ijscr.2023.109059

**Published:** 2023-11-13

**Authors:** Romy Deviandri, Dhandia Rifardi

**Affiliations:** a Department of Orthopedics, University of Groningen, University Medical Center Groningen, Groningen, the Netherlands; b Department of Physiology-Faculty of Medicine, Universitas Riau, Pekanbaru, Indonesia; c Department of Surgery, Division of Orthopedics, Faculty of Medicine, Universitas Riau, Arifin Achmad Hospital, Pekanbaru, Indonesia

**Keywords:** DRUJ, Wrist instability, Ligament reconstruction, Palmaris longus, Suture anchor

## Abstract

**Introduction:**

The chronic instability of the DRUJ should be appropriately treated. Ligament reconstruction in the original technique needs an adequate length of the graft, which needs to be modified in such a case.

**Case presentation:**

A 27-year-old male presented with right wrist pain accompanied by limited movement that has been felt for the last two months. There was an obvious deformity with tenderness. Palpation revealed a positive ballottement and piano-key sign test. An X-ray examination revealed a union fracture one-third distally on the right radius bone with dorsal dislocation of the right distal radioulnar joint. The result of an MRI confirmed a triangular fibrocartilage complex tear. The patient was diagnosed with chronic DRUJ instability.

**Discussion:**

We performed a chronic DRUJ reconstruction using the harvesting palmaris longus tendon. However, the length of the graft is too short. Further, we performed a modified technique with suture anchor fixation for this patient. This technique could be a helpful alternative if the length of the graft is insufficient. As a result, there was an improvement in the DASH score and EQ5D questionnaires.

**Conclusion:**

Chronic DRUJ instability could be treated by ligament reconstruction with modified suture anchors fixation in the inadequate length of the graft situation.

## Highlights

• The chronic instability of the DRUJ could be appropriately treated by ligament reconstruction.
• The ligament reconstruction of DRUJ in the original technique needs an adequate length of the graft, which needs to be modified in such a case.
• A chronic DRUJ could be treated by ligament reconstruction with modified suture anchors fixation in the inadequate length of the graft situation.

## 1 Introduction

The stability of the distal radioulnar joint (DRUJ) results from the bony structure and the integrity of the surrounding soft tissues, including the triangular fibrocartilage complex (TFCC), pronator quadratus, and interosseous membrane. The dorsal and palmar radioulnar ligaments are considered significant factors of DRUJ stability, whereas the bony structure accounts for only 20 % [1]. DRUJ instability is typically defined by increased translation of the ulna relative to the radius compared with the contralateral wrist. DRUJ instability is common but often misdiagnosed. This instability is common in the setting of distal radius fractures [2]. Untreated instability changes the kinematics of the wrist and forearm, which can result in pain, weakness, and possibly degenerative arthritis [3].

Reconstruction of the distal radioulnar ligaments offers the best possibility of restoring normal DRUJ primary constraints and kinematics [4,5]. When reconstructing ligaments using the method described by Adam and Berger in 2002, the palmaris longus is the preferred tendon donor [3,4]. However, sometimes, we found the graft length too short. A modified technique is needed in this particular case to get satisfactory results [6]. In this case report, we will present a case of chronic instability of the DRUJ in our hospital with a previous history of distal radius fractures, which was managed by ligament reconstruction using the harvesting palmaris longus and modified suture anchor technique, and describe the follow-up after treatment. This study is reported in line with the SCARE 2020 guidelines [7].

## 2 Case presentation

A male, 27 years old, right-handed, came with right wrist pain accompanied by limited movement that has been felt for the last two months. Previously, the patient had a history of radius fractures due to a fall from 2 m high while repairing the tile. The patient had a plate installed on May 18th, 2021, and removed on May 20th, 2022. After the implant removal, the symptoms appeared significantly. The patient had no other medical condition, medication, or allergy. Before being injured, the patient worked as an office worker.

On physical examination of the right wrist, we found obvious deformity with minimal tenderness. Palpation revealed a positive ballottement and piano-key sign test. There were limitations in the range of movement, which showed a 10–15^0^ of flexion, 40^0^ of supination, and 30^0^ of pronation. When compared to the contralateral limb and the right upper extremity were within normal limits.

**Fig. 1:**
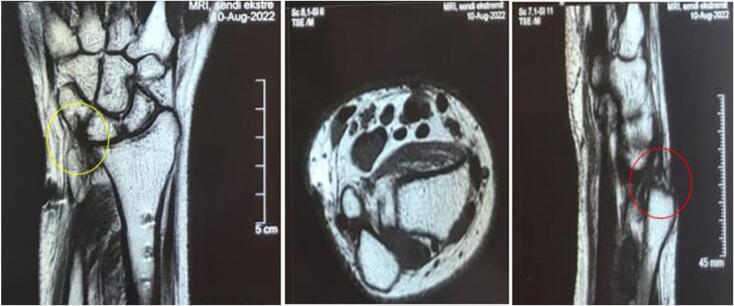
Initial right forearm X-ray examination after implant removal when DRUJ being symptomatic.

**Fig. 2:**
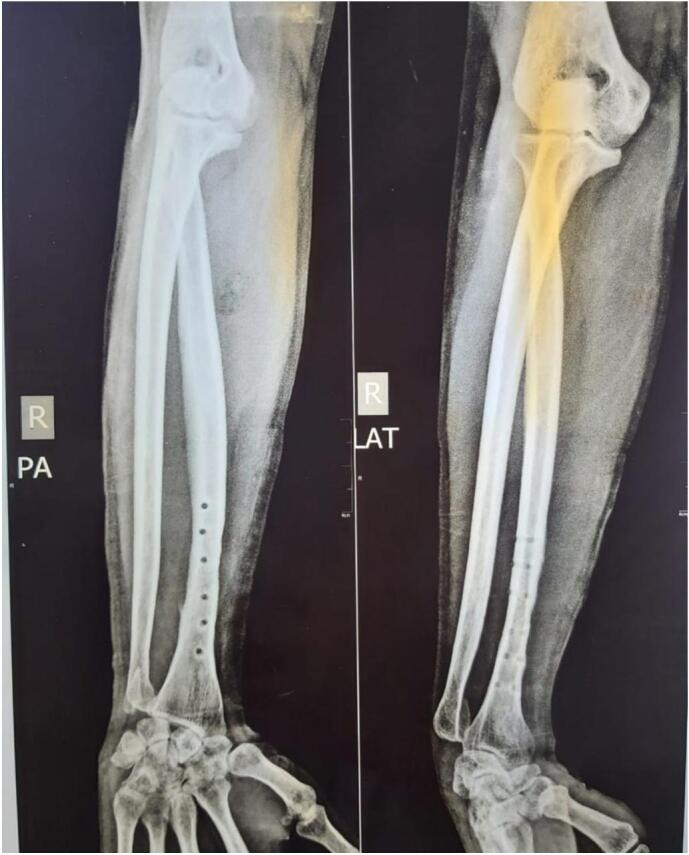
Preoperative MRI of the right wrist in coronal (a), axial (b), and sagittal (c) planes showing a dorsal dislocation of the right distal radioulnar joint and a triangular fibrocartilage complex tear.

An X-ray examination of the right forearm revealed a union fracture one-third distally on the right radius bone with dorsal dislocation of the right distal radioulnar joint (Fig. 1). We continued the test with an MRI without contrast which confirmed a triangular fibrocartilage complex tear (Fig. 2). Based on physical examinations and radiological imaging, we diagnosed this patient as having a chronic DRUJ instability.

**Fig. 3:**
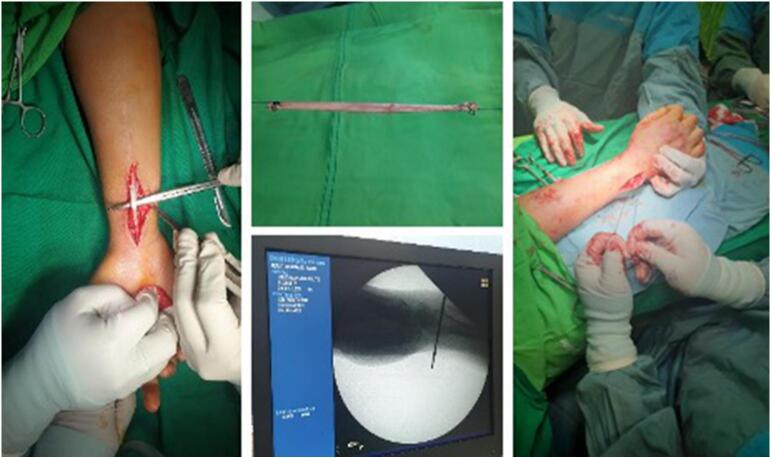
Surgical step of DRUJ reconstruction. Harvesting palmaris longus tendon following ligament reconstruction in chronic instability DRUJ (a). The harvested 10 cm long tendon (b). Lateral X-ray demonstrating guide wire in distal radius (c). The graft was routed on the distal radius from posterior to anterior, then routed to the distal ulna in the fovea (d).

**Fig. 4:**
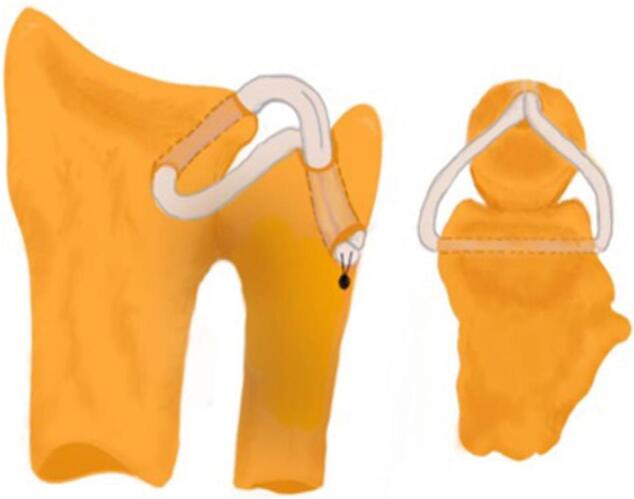
The schematic picture of DRUJ reconstruction showed the harvested palmaris longus fixed the DRUJ instability through bone tunnels, then the fixation using 1.5 mm suture anchors as the length of the graft is insufficient.

As this is a chronic case and the patient is actively working, it is recommended to do surgery with the DRUJ ligament reconstruction technique [[3], [4], [5]]. The surgery was performed on September 9th, 2022, after consent. The following are the surgical steps: (1) the patient was in a supine position with general anaesthesia, (2) the dorsal and palmar wrist was incised, (3) a palmaris longus tendon with a diameter of 4,5 mm was harvested, (4) the graft was routed on a distal radius from posterior to anterior, then routed to the distal ulna in the fovea, (5) because of the length of the palmaris longus tendon was relatively short for performing the standard loop around the neck of ulna, less than 10 cm in total, it was fixed on the ulnar site by a 1.5 mm suture anchor (Soft anchor, Doratek), 15 mm below the ulnar tunnel. The appropriate awl was used to prepare an anchor hole, and then, when the proper hole depth was achieved, the specified anchor was located to fix the graft suitably (Fig. 3, Fig. 4), (6) Then, the wound was sutured [3,4].

After surgery, the forearm patient wore a long arm cast in a neutral to slight supination position extending to just above the elbow to control forearm rotation, which was removed in the first month after surgery. The patient was sent to physical therapy to regain his functional range of motion. Therapy began with active and gentle passive wrist flexion, extension, pronation, and supination. No limitations are placed on active motion, but only gentle passive motion should be used during the first month of therapy. Three months after surgery, strengthening exercises were initiated, although excessive forces were avoided when the arm was fully in supination or pronation [4].

**Fig. 5:**
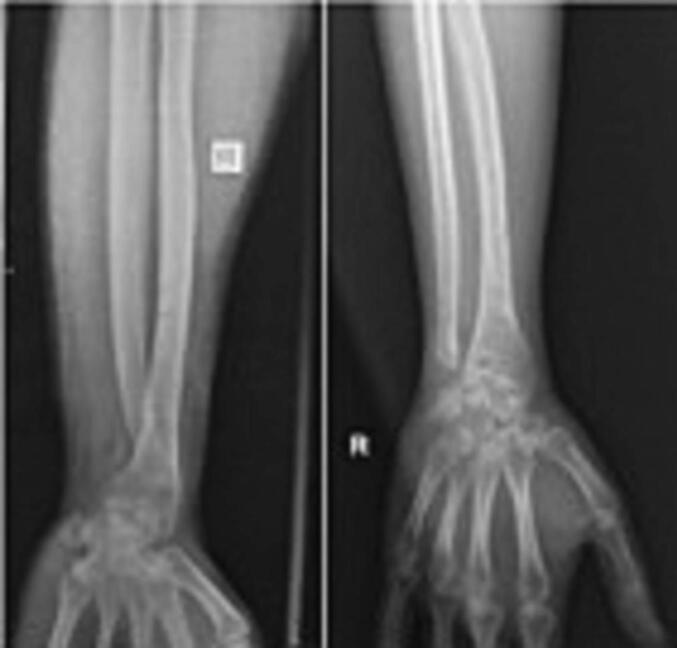
Right forearm X-ray examination before (left) and after (right) surgery shows the improvement of the DRUJ.

In the third month after surgery, the patient could perform daily activities such as turning a key, writing, and washing his hair and back. However, the patient still found it difficult to perform heavy tasks such as carrying a heavy object (weighing more than 10 pounds), gardening or pushing open a heavy door. The patient's right wrist is still sore and stiff but improving. At four months postoperatively, more aggressive passive ROM and strengthening are added [3]. In the six months after surgery, the patient was able to perform daily activities, from light to heavy tasks, and an X-ray result of the right forearm was normal (Fig. 5). From the sixth month of follow-up, a good outcome is also achieved based on the results of the DASH Score and EQ5D questionnaires. The DASH Score increased from 65.8 before surgery to 1.7, and the EQ5D score went from 0.64 before surgery to 0.92.

## 3 Discussion

This case report aims to assess patient outcomes from chronic DRUJ reconstruction using the harvesting palmaris longus and modified suture anchor technique because of the insufficient length of the graft. It shows a good outcome for this patient after undergoing this reconstruction and rehabilitation program six months after surgery. A good outcome is achieved based on the results of the DASH score and EQ5D questionnaires.

Clinicians have used various modalities for the diagnosis of TFCC. Besides clinical tests, MRI and Computed Tomography (CT) with or without angiography are useful imaging for evaluating a TFCC. A computational simulation was also helpful in some cases [3,4,8]. Further, a growing body of knowledge proves that arthroscopy and open findings of surgery are still considered a gold standard [8]. However, because of the limited resources in our centre, we have not performed arthroscopic procedures yet.

Various surgical techniques have been described in the literature regarding DRUJ treatment [9]. Delay direct repair of the TFCC is the initial surgical option. Reconstructive treatment is necessary when the TFCC cannot be repaired due to primary tissue damage or retraction. Three categories can be used to group the numerous reconstructions that have been described: (1) a direct radioulnar tether that is extrinsic to the joint, (2) an indirect radioulnar link via an ulnocarpal sling or tenodesis, and (3) reconstruction of the radioulnar ligaments. Even though the methods in the first two categories may reduce symptoms, they are not anatomic, and laboratory studies indicate that they do not restore normal joint stability or mechanics of the DRUJ [4,9]. The palmaris longus (PL) is the preferred tendon donor for ligament reconstruction. On the other hand, it may display anatomical alterations, such as agenesis and changes in morphology, position, and attachment [5]. In this case report, we performed a chronic DRUJ reconstruction using the harvesting palmaris longus technique. But intraoperatively, we found PL was relatively short for performing the standard loop around the neck of the ulna. So, we decided to use suture anchors for fixation. A suture anchor would provide adequate mechanical fixation [6,10]. Alternatively, interference screw fixation could be used either antegrade or retrograde [11].

Various techniques are mentioned in the literature in the event of a persistent instability of DRUJ. The restoration of total dislocation by tendon loops proximal to the DRUJ is described using standard Adam-Berger or with modified methods. The osseous pathology of the DRUJ's instability is addressed by the restoration of the sigmoid notch. In a clinical instance, sigmoid notch osteotomy is reported to be successful in reconstructing a ligament that had failed earlier. In the gross ligamentous instability caused by insufficient ulnar insertion of the TFCC and partial rupture of the distal interosseous membrane of the forearm, Gabl et.al showed a rotation osteotomy of the distal ulna as a reconstructive procedure for chronic dislocations of the DRUJ was helpful [12].

DRUJ reconstruction is considered a relative contraindication in the situation of DRUJ arthritis due to its potential to increase joint contact force, hence exacerbating the arthrosis. Even yet, reconstruction may still be tried on active arthritic patients who are unlikely to benefit much from a salvage procedure—especially since a salvage may be necessary if the reconstruction fails. If a salvage operation is an option, Sauve Kapandji or the Darrach procedure may be used [13,14].

Complications following reconstruction for DRUJ instability are persistent joint pain, recurrent instability, stiffness, and weakness [4]. The harvesting palmaris longus technique can increase the risk of median nerve injury [15]. In this case, the patient still complains of pain and stiffness in the right wrist three months after surgery. Because of that, the patient is unable to do any heavy activity. For the subsequent follow-up, we have to train and strengthen the muscles to increase function and the quality of life. Strengthening is started three months postoperatively, but high forces with the arm in full pronation or supination are avoided. At four months postoperatively, more aggressive passive ROM and strengthening are added, with the goal of recovering 85 % of normal forearm rotation by six months. No unprotected use of the hand for sports or lifting more than 10 lbs. is permitted until at least four months after surgery [4]. Six months after surgery, the patient could perform daily activities, as evidenced by the improvement in his DASH score and EQ5D questionnaires. This result was comparable with other methods in the literature, such as by interference screw fixation or the standard loop around the ulnar neck. This is supported by Gillis et al. who showed no difference in success rate among procedures [16]. However, the anchor technique is beneficial in terms of the inadequate length of the graft.

## 4 Conclusion

Chronic DRUJ instability could be treated appropriately by ligament reconstruction surgery using a palmaris longus tendon and modified fixation with suture anchors. The improvement was demonstrated by decreased pain symptoms, decreased ulnar subluxation, improved DASH score, and improved EQ5D questionnaires. It should be considered to recover a DRUJ instability by ligament reconstruction with modified suture anchors fixation in the inadequate length of the graft situation.

## Provenance and peer review

Not commissioned, externally peer-reviewed.

## Consent

Written informed consent was obtained from the patient to publish this case report and accompanying images. A copy of the written consent is available for review by the Editor-in-Chief of this journal on request.

## Ethical approval

Non-applicable.

This study is exempt from ethical approval in my institution because this study is based on the daily clinical practice of the author in the institution instead of an experiment study.

## Funding

This case report did not receive any specific grant from public, commercial, or not-for-profit funding agencies.

## Author contribution

The first author (RD) contributed to the study concept or design, data analysis or interpretation, and paper writing.

The second author (DR) contributed to the study concept or design, data collection, data analysis or interpretation, and revising the paper.

## Guarantor

The first author (RD) is the guarantor for this study.

## Research registration number

Non-applicable.

This study is not first in man.

## Conflict of interest statement

The authors have no conflicts of interest to declare.

## Appendix A Supplementary data

Fig. 6 MRI T1-T2 sequences of the wrist joint from the axial, coronal, and sagittal view showed the TFCC tear. Fig. 6
