# Supplementary material for: Ligament reconstruction with modified suture anchor fixation technique for chronic distal radioulnar joint instability: A case report and literature review
Source: Int J Surg Case Rep. 2023 Nov 13;113:109059. doi: 10.1016/j.ijscr.2023.109059 (PMC10684790; doi:10.1016/j.ijscr.2023.109059)
Supplement: Fig. 6 — MRI T1-T2 sequences of the wrist joint from the axial, coronal, and sagittal view showed the TFCC tear. [file mmc1.pdf]

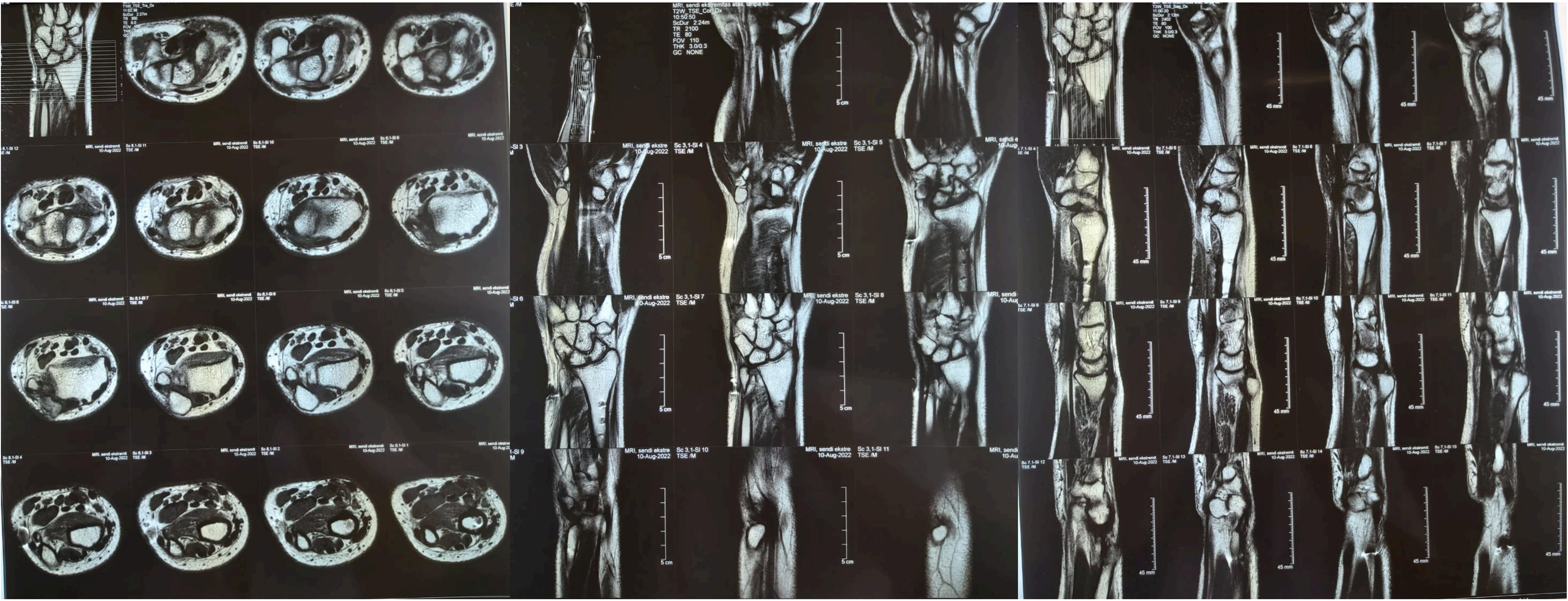

MRI, sendi ekstremitas atas, tanpa ko...
T2W_TSE_Cor_Dx
10:50:50
ScDur 2:24m
TR 2100
TE 80
FOV 110
THK 3.0/0.3
GC NONE
Sc 3,1-Sl 4
TSE /M
MRI, sendi ekstre
10-Aug-2022
Sc 3,1-Sl 5
Sc 3,1-Sl 7
Sc 3,1-Sl 8
Sc 3,1-Sl 10
Sc 3,1-Sl 11
5 cm
45 mm
